# Signalling through Src family kinase isoforms is not redundant in models of thrombo‐inflammatory vascular disease

**DOI:** 10.1111/jcmm.13721

**Published:** 2018-07-04

**Authors:** Matthew J. Harrison, Myriam Chimen, Mohammed Hussain, Asif J. Iqbal, Yotis A. Senis, Gerard B. Nash, Steve P. Watson, G. Ed Rainger

**Affiliations:** ^1^ Institute of Cardiovascular Sciences College of Medical and Dental Science The Medical School University of Birmingham Birmingham UK

**Keywords:** atherosclerosis, inflammation, monocytes, platelets, Src family kinases

## Abstract

The Src family kinases (SFK) are a group of signalling molecules with important regulatory functions in inflammation and haemostasis. Leucocytes and platelets express multiple isoforms of the SFKs. Previous studies used broad‐spectrum pharmacological inhibitors, or murine models deficient in multiple SFK isoforms, to demonstrate the functional consequences of deficiencies in SFK signalling. Here, we hypothesized that individual SFK operate in a non‐redundant fashion in the thrombo‐inflammatory recruitment of monocyte during atherosclerosis. Using in vitro adhesion assays and single SFK knockout mice crossed with the ApoE^−/−^ model of atherosclerosis, we find that SFK signalling regulates platelet‐dependent recruitment of monocytes. However, loss of a single SFK, Fgr or Lyn, reduced platelet‐mediated monocyte recruitment in vitro. This translated into a significant reduction in the burden of atherosclerotic disease in *Fgr* ^−/−^ */ApoE* ^−/−^ or *Lyn* ^−/−^ */ApoE* ^−/−^ animals. SFK signalling is not redundant in thrombo‐inflammatory vascular disease and individual SFK may represent targets for therapeutic intervention.

## 1 INTRODUCTION

Primary atherosclerosis is a chronic inflammatory, fibro‐proliferative disease of the artery wall, in which arterial plaques of great cellular and molecular complexity develop over many years.^1^ The recruitment of inflammatory leucocytes from the blood into the artery wall occurs at all stages of the disease and this process is widely assumed to be dependent upon the well‐documented multi‐step paradigm of leucocyte trafficking that supports leucocyte recruitment in the post‐capillary venules during acute, resolving inflammatory responses.^2^ However, in atherosclerosis there is strong evidence that alternative thrombo‐inflammatory pathways of leucocyte trafficking are provided by interactions between platelets, leucocytes and vascular endothelial cells.^3^, ^4^, ^5^, ^6^ In the context of the therapeutic control of inflammation in atherosclerosis, these alternative pathways are important, as they bypass the usual regulatory mechanisms that control the magnitude and duration of the inflammatory response. This makes it difficult to target leucocyte recruitment in atherosclerosis using current anti‐inflammatory strategies. However, the provision of distinct molecular and signalling pathways that support platelet‐mediated leucocyte accumulation in atherosclerosis provides an opportunity to develop new, disease specific, drugs.

The expression of P‐selectin on activated platelets is reported to be important for establishing adhesive contact with circulating leucocytes.^7^ Thus, an obvious target for intervening in thrombo‐inflammatory leucocyte trafficking is the inhibition of platelet activation. However, patients at high risk of cardiovascular disease are already well served by antiplatelet agents such as aspirin and the antagonists of the ADP receptor P2Y12 (eg Prasugrel and Ticagralor).^8^, ^9^, ^10^ Although these show anti‐inflammatory activity over and above their ability to prevent thrombosis,^11^, ^12^ their use does not prevent progression of atherosclerosis in humans.^13^ Thus, we require a different approach to target thrombo‐inflammatory pathways with greater precision. We believe that targeting signalling pathways involved in the activation and function of both platelets and leucocytes represent tractable targets for such interventions, and the Src family kinases (SFKs) may be appropriate due to their importance in regulating the function of both cell types.

In mammals, SFKs are a group of 8 structurally related protein tyrosine kinases.^14^ Src, Fyn and Yes are ubiquitously expressed,^15^ although distinct isoforms dominate expression in different cells.^16^, ^17^, ^18^ Lyn, Hck, Fgr, Blk and Lck however, are predominantly and differentially expressed in the various cells types of the haematopoietic lineage, ie leucocytes and platelets.^19^ Leucocyte SFKs play an important role in the regulation of many activation responses, but they are considered particularly important during the development of an inflammatory response. In particular, leucocyte recruitment by cytokine‐activated endothelial cells, resulting in integrin‐mediated leucocyte activation and migration in to tissue, is an essential process in the inflammatory response. Although non‐redundant functions of Fyn have been identified in neuronal maturation and spatial learning in knockout animals,^20^, ^21^ leucocytes deficient in multiple SFK have been presumed necessary to remove redundant SFK signalling during leucocyte recruitment in inflammation. Such a strategy inhibits the function multiple adhesion pathways, including the β1 and β2 integrins essential for leucocyte trafficking.^22^, ^23^, ^24^ In addition, leucocyte functions that may be dependent on outside‐in signals originating from ligand bound integrins are also affected in SFK‐deficient animals.^25^, ^26^ For example, initiation of the respiratory burst, degranulation and phagocytosis are known to be impaired in the absence of SFK signalling.^27^, ^28^ SFK are also essential for platelet activation. They mediate rapid activation down stream of adhesion receptors such as GPIb‐IX‐V (von Willebrand receptor^29^) and GPVI (collagen receptor^30^), they promote maximal platelet activity by signalling downstream of G‐coupled receptors for secondary mediators such as ADP,^31^ and they regulate outside‐in signals from extra‐cellular matrix binding integrins such αIIbβ3, required for efficient activation, spreading, secretion and clot retraction.^32^

Importantly however, the roles of SFK in thrombo‐inflammatory pathways of leucocyte recruitment have not been investigated to date. Here, we show that the platelet‐dependent recruitment of monocytes in in vitro models of vascular inflammation was strongly dependent upon SFK's in both murine and human systems. Using genetically modified mice in which the most (Lyn) and least (Fgr) abundant platelet SFK isoforms had been knocked out independently, the platelets of both Fgr^−/−^ and Lyn^−/−^ deficient animals showed greatly retarded activation responses to ADP in vitro. When these platelets were used in functionally stringent thrombo‐inflammatory models of leukcoyte recruitment in vitro, both strains showed significant deficiencies in leucocyte recruitment. We also observed dramatic reductions in the burden of disease in the Fgr^−/−^/ApoE^−/−^ and Lyn^−/−^/ApoE^−/−^ models of atherosclerosis.

## 2 MATERIALS AND METHODS

### 2.1 Animals

*Fgr* ^−/−^ *ApoE* ^−/−^ and *Lyn* ^−/−^ *ApoE* ^−/−^ mice on a C57BL6 background were generated from *ApoE* ^−/−^ (Charles River), *Lyn* ^−/−^ (Jackson Laboratoy) and *Fgr* ^−/−^ (Prof Clifford Lowell, UCSF, San Francisco, USA) animals. All mice were maintained at the Biomedical Services Unit at the University of Birmingham, according to Home Office regulations. Mice were genotyped using the DNeasy blood and tissue kit (Qiagen, Germany), according to manufacturer's instructions. Age and sex matched *Fgr* ^−/−^ *ApoE* ^−/−^, *Lyn* ^−/−^ *ApoE* ^−/−^ and *ApoE* ^−/−^ mice were maintained on chow diet between weaning and 6 weeks. At 6 weeks, the mice were placed on a high fat diet (HFD) (21.4% cocoa butter [w/w] and 0.2% cholesterol [w/w]; Special Diet Services, UK) for 6 weeks. We observed no sex differences in our analysis so both male and female data were included.

### 2.2 Murine blood collection for in vitro studies

Murine blood was drawn by cardiac puncture from mice terminally anesthetized with CO_2_ and taken into 100 μL sodium citrate and combined with 200 μL modified Tyrode buffer (137 mmol/L NaCl. 11.9 mmol/L NaHCO3, 0.4 mmol/L Na2HPO4, 2.7 mmol/L KCl, 1.1 mmol/L MgCl2, 20 mmol/L Hepes and 5.6 mmol/L glucose, pH 7.3). Blood was left at room temperature until further processing.

### 2.3 Murine hematological and serum lipid analysis

Total white blood cell (WBC) counts and differential white cell counts were determined using the ABX Pentra 60 hematology analyser (Horiba, Northampton, UK). Non‐anticoagulated blood was allowed to clot at room temperature for 1 hour then centrifuged at 20 000 ***g*** for 10 minutes. Serum was collected and stored at −20°C. Serum triglyceride (Randox, Antrim, UK), free fatty acids (Abcam, Cambridge, UK), total cholesterol levels, HDL, LDL and glucose (Sentinel Diagnostics, Milan, Italy) were analysed via colorimetric assays.

### 2.4 Murine aorta isolation

Mice were sacrificed via terminal intraperitoneal (IP) injection of 200 mg/kg Pentobarbital. Aortas were fixed in situ by the perfusion of 2% paraformaldehyde (Sigma, UK) through the left ventricle of the heart. The whole aorta was excised, cut longitudinally and stained with Oil Red O (ORO; Sigma). Digital photographs were taken and analyzed for lesion size, as a percentage of a specific region (whole aorta, inner and outer curvature, aortic arch, aortic branches, thoracic and abdominal aorta), using ImageJ software (NIH, USA). Detailed analysis of plaque burden in the descending aorta was conducted in order to evaluate the total number and average size of plaques in an area of the aorta which has several branch points (intercostals, mesenteric and renal arteries). Digital images were analysed in ImageJ software (NIH) via threshold analysis. Individual plaques were analysed with a threshold of 0.5 mm^2^ and circularity of 0.04 mm.

### 2.5 Histology and immunofluorescence microscopy of atherosclerotic lesions in frozen sections

Lesion stability and morphology was assessed in OCT‐embedded frozen aortic root sections, which had been previously perfusion fixed with 4% paraformaldehyde. The aortic root was sectioned at −20°C at 7 μm per section. To quantify collagen content sections were stained with van Gieson's (Merck, Germany) according to the manufacturer's instructions. Staining with DAPI was also utilized to quantify cellular content within the plaque. All images were analysed with ImageJ software via threshold analysis. Van Gieson stained aortic arch sections were imaged using the Axio ScanZ1 (Zeiss, Germany) at ×20 magnification and DAPI‐stained sections were imaged using the Olympus BX61 Upright Motorized Microscope (Olympus, Japan) at ×4 magnification. Plaque collagen content and cellularity analysis was carried out using Fiji (NIH). Once imaged, tissue sections had masks applied over plaques within the aortic arches and the area quantified before and after applying a colour threshold selection for red pixels (red denotes collagen positivity using Van Gieson). Total plaque area pixel counts and total plaque red pixel counts were used to calculate % collagen positivity within plaques. In order to quantify the cellularity within plaques, the integrated density of DAPI staining was measured after manual selection of plaques within aortic arch sections from ApoE^−/−^, ApoE^−/−^Lyn^−/−^ and ApoE^−/−^Fgr^−/−^ mice.

### 2.6 Human blood collection for in vitro studies

Venous blood from healthy volunteers was taken into 10% sodium citrate or EDTA.

### 2.7 Preparation of platelet rich plasma

Platelet‐rich plasma (PRP) was obtained from human and murine anti‐coagulated blood by addition of modified Tyrode's buffer 1:5 to volume of whole blood and centrifugation at 300× *g* for 10 minutes. PRP was then removed and diluted to the required concentration with Tyrode's/Hepes buffer.

### 2.8 Peripheral blood mononuclear cell isolation

Peripheral blood mononuclear cells (PBMCs) were isolated from human EDTA treated blood using the histopaque 1119 and 1077 gradient system (Sigma). Murine PBMCs were isolated using Lympholyte Mammal (Cedarlane Laboratorys). Briefly, 2 mL of EDTA treated Blood was diluted 1:1 with PBS and layered over 5 mL of Lympholyte mammal and centrifuged at 800× *g* for 20 minutes at room temperature. PBMC layer was removed and diluted to 10 mL with PBS and centrifuged at 800× *g* for 10 minutes at room temperature to pellet cells. Supernatant was removed and PBMCs were diluted to 1 × 10^6^ cells/mL in PBS.

### 2.9 Aggregometry

Platelet aggregation was monitored in 300 μL (2 × 10^8^ /mL) PRP. Aggregation was monitored by light transmission using a Born aggregometer (Alpha Laboratories, Eastleigh, Hants, UK) with high‐speed stirring (1200 rpm) at 37°C. Agonists were added as 10‐100‐fold concentrates. The transmission with PRP was expressed as a percentage of that with platelet‐poor plasma.

### 2.10 Flow cytometry analysis of P‐selectin expression

Platelet‐rich plasma was extracted as previously described. 5 μL PRP was added to 45 μL Tyrode's buffer. Platelets were incubated with 5 μL CD41 Allophycocyanin (APC), (Human‐BD, 559777 & Mouse‐ebioscience, 17‐0411‐82) and CD62P Fluorescein (FITC), (Human‐BD, 555523 & Mouse‐Emfret, M130‐1) and incubated at 37°C for 30 minutes ± 30 μmol/L ADP, Dasatinib (LC Laboratories, Woburn, MA, USA) and/or 0.1% DMSO. The reaction was then stopped with the addition of 1% ice cold formalin and analysed on a BD Accuri using C6 software (version 1.0.264.21).

### 2.11 Human flow‐based adhesion assay on VWF

Blood was collected as previously described. Capillary tubes^1^ (0.1 × 1.0 mm, 50 mm long; Camlab, Cambridge, UK) were coated with 0.1 mg/mL human Von Willebrand factor (VWF) (HTI, Vermont, USA‐HCVWF‐0190) overnight at 4°C. The capillaries were washed and blocked with PBS containing 2% BSA (PBSA) for 2 hours at room temperature. Capillaries were then rinsed with PBSA, and connected to a multi‐valve flow‐based system containing reservoirs filled with either anti‐coagulated blood, PBMCs or PBSA. The anti‐coagulated blood was perfused through the VWF‐coated microcapillary at a shear rate of 1000^−s^ for 2 minutes. Capillaries were then washed with 0.1% PBSA (±30 μmol/L ADP) at 1000^−s^, before PBMCs were perfused. Platelet/monocyte adhesion was monitored at 100^−s^ by phase contrast microscopy. At least 6 different microscope fields (20× objective) were analysed. Image analysis was performed off‐line using ImagePro plus software (DataCell Limited, Berkshire, UK).

### 2.12 Murine flow‐based adhesion assay on VWF

Blood was collected as previously described. Capillary tubes (0.1 × 1.0 mm, 50 mm long; Camlab) were coated with 3.1 g/L anti‐Human VWF (Dako, Cambridge, UK—A0082) overnight at 4°C. The capillaries were washed and blocked with PBS containing 2% BSA for 1 hour at room temperature, followed by murine plasma for 1 hour at room temperature. Capillaries were then connected to a multi‐valve flow‐based system containing reservoirs filled with either anti‐coagulated blood, PBMCs or PBSA. The anti‐coagulated blood was perfused through the VWF‐coated microcapillary at a shear rate of 1000^−s^ for 2 minutes. Capillaries were then washed with PBSA (±30 μmol/L ADP) at 1000^−s^, before PBMCs were perfused. Platelet/monocyte adhesion was monitored at 100^−s^ by phase contrast microscopy. At least 6 different microscope fields (20 × objective) were analysed. Image analysis was performed off‐line using ImagePro plus software (DataCell Limited).

### 2.13 Isolation and culture of Human Endothelial cells

Human umbilical vein endothelial cells (HUVEC) were isolated and characterized as described previously.^33^ Each experiment used first passage ECs from a different donor and cells were cultured on APES‐coated glass capillary microslides. EC cultures in microslides were either untreated or stimulated with 10 ng/mL TGF‐β1 for 24 hours.

### 2.14 Reagents

Dasatinib (LC Laboratories) was dissolved in dimethyl sulfoxide (DMSO) at a stock concentration of 1 mmol/L. Final concentrations of 20 μmol/L (0.1% DMSO) were used in all experiments. DMSO was included in all assays as a solvent control.

### 2.15 Statistical analysis

Experimental data were analysed using GraphPad Prism software (GraphPad, La Jolla, USA‐version 5) or SPSS (IBM). Normality of data was checked using the Shapiro‐Wilk normality test when n ≤ 10 or the D'Agostino & pearson normality test when n ≥ 10. Non‐parametric tests were used when the data did not pass the normality tests. Differences between individual treatments or groups were analysed by paired or unpaired *t* test as appropriate. One or two‐way analysis of variance (ANOVA) was used for multiple group comparison followed by a post hoc analysis where appropriate, using Bonferroni test for comparisons between groups or Dunnet tests for comparisons to a control group. *P* values of ≤.05 were considered significant. Data are expressed as mean ± standard error of the mean (SEM) when n ≥ 8 or standard deviation (SD) when n ≤ 8.

### 2.16 Study approval

Animal studies were conducted under Home Office licence PPL 400 3659. Samples from healthy human volunteers were conducted following ethical approval (Birmingham University Research Ethics Committee, Birmingham, UK) and according to the Declaration of Helsinki Principles. All participants gave their written informed consent before samples were taken.

## 3 RESULTS

### 3.1 SFK are required for thrombo‐inflammatory recruitment of leucocytes in in vitro models of vascular inflammation

**Figure 1:**
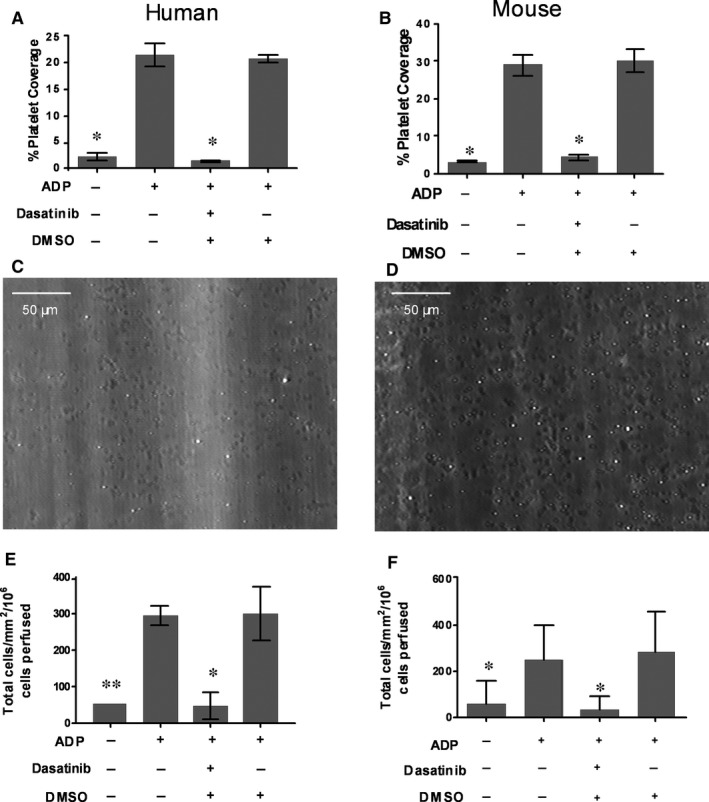
Dasatinib inhibits platelet activation and recruitment of monocytes on immobilized VWF under flow conditions. A, Percentage platelet coverage from flowing human whole blood (1000^−s^) on a matrix of immobilized VWF in the presence of 30 μmol/L ADP ± 20 μmol/L broad spectrum SFK inhibitor, (B) or using murine whole blood (n = 3). C, Representative images of human or (D) murine platelet adhesion to VWF in the presence of 30 μmol/L ADP under flow conditions (1000^−s^). E, Total adhesion of Human or (F) murine monocytes in the presence of 30 μmol/L ADP ± 20 μmol/L Dasatinib under flow conditions (100^−s^). The level of monocyte and platelet adhesion was assessed by phase contrast microscopy (n = 3). Data are shown as mean ± SD. *P < .05, **P < .01 compared to the ADP positive control using one‐way ANOVA followed by a Dunnett's post‐test

Studies using broad spectrum inhibitors of SFK function, such as Dasatanib or PP2, show that removal of redundant SFK signalling can efficiently ablate the activation of platelets using *in vitro* assays such as platelet aggregometry.^31^ Here, we confirm that Dasatnib is an efficient inhibitor of the aggregation of both human and murine platelets (Supporting information Figure S1A‐F). The loss of single SFK isoforms using genetically altered strains of mice (specific inhibitors have yet to be developed to allow pharmacological intervention in a SFK isoform‐specific manner) presents a different picture. Uniformly, such studies report little if any effect of the loss of single SFK isoforms on platelet function in aggregometry assays, and the animals present with a mild, if any, bleeding diathesis, when the haemostatic system is tasked in vivo.^32^, ^33^ However, studying the aggregation of purified platelets in stirred suspensions has no physiological correlate, and induction of efficient aggregation requires high levels of agonist. Indeed, in our own experiments we saw little effect of the genetic ablation of the single SFK isoforms, Lyn or Fgr, when platelet responses were tested in aggregomtery experiments using 10 or 30 μg/mL ADP. However, when we titrated ADP below such concentrations (1‐5 μg/mL) we were able to see subtle but significant deficiencies in the aggregation responses of platelets isolated from either knockout strain (Supporting information Figure S2A,B). The fact that differences in platelet function were discernible in aggregometry experiments immediately prompted a detailed analysis in stringent flow‐based adhesion assays using physiological substrates for platelet recruitment. When CPDA anti‐coagulated whole blood was perfused at a wall shear rate of 1000 s^−1^ across VWF immobilized in glass microslides, a large population of rapidly rolling platelets was established. This was the case for human blood perfused across hVWF (Figure 1A) and for murine blood (Figure 1B) perfused across mVWF. These interactions were not stable and after washout of blood, few platelets remained on the adhesive surface (Figure 1A,B). However, by adding 30 μmol/L ADP to the wash buffer, adhesion was efficiently stabilized when the platelets were activated and spread on the adhesive substrate to provide between 20% and 30% coverage (Figure 1A‐D). When a bolus of isolated monocytes (or mononuclear cells in the murine system) was perfused (at 100 s^−1^) over unactivated platelets there was little leucocyte adhesion, demonstrating that VWF was not a substrate that could promote efficient recruitment of flowing monocytes in the absence of activated platelets (Figure 1E,F). In contrast, there was substantial secondary recruitment of monocytes to ADP activated platelets as these cells provided an adhesive bridge between the flowing leucocytes and the VWF substrate (Figure 1E,F).

When these experiments were conducted in the presence of Dasatinib (20 μmol/L), platelet activation and adhesion were effectively abolished (>90% inhibition in human and murine‐based assays; Figure 1A,B). Ablation of redundant SFK signalling in platelets had a dramatic effect on the secondary recruitment of monocytes. Thus, in both the human and murine systems, monocyte adhesion was reduced by ≈90%, which paralleled the loss of platelet coverage (Figure 1E,F). The loss of monocyte adhesion to the extent observed was surprising, as we and others have previously shown that even sparsely adherent platelets (ie <1% coverage) can support substantial levels of leucocyte tethering and adhesion through P‐selectin.^6^, ^34^ In addition, using this flow system, we observed that efficient recruitment of human neutrophils on platelets bound to VWF (Supporting information Figure S3). Treatment of whole blood with Dasatinib (20 μmol/L) also significantly decreased neutrophil adhesion by 89%.

**Figure 2:**
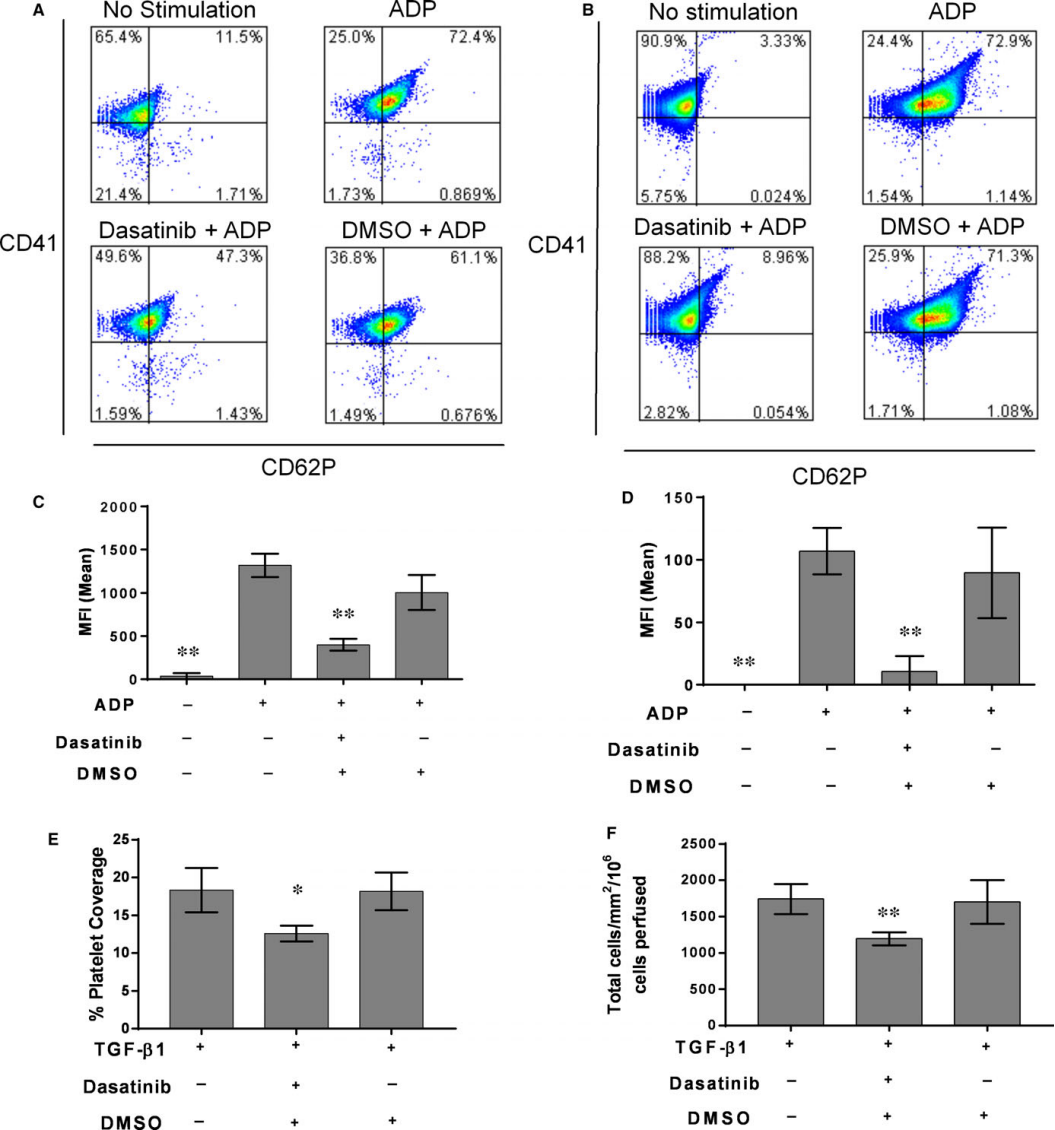
Dasatinib inhibits platelet P‐selectin expression in both human and mouse platelets and prevents the adhesion of platelets and monocytes to TGF‐β1 stimulated endothelium under flow conditions. A, Representative flow cytometry plots of P‐selectin expression in human or (B) murine platelet‐rich plasma (PRP) prepared from freshly drawn blood and stimulated with 30 μmol/L ADP ± 20 μmol/L Dasatinib. C, Mean fluorescent intensity (MFI) of P‐selectin staining on human or (D) murine platelets. E, The effect of Dasatanib on platelet and (F) monocyte recruitment to TGF‐β1 stimulated endothelial cells. Dasatanib (4 μmol/L) was added to blood 15 min prior to perfusion across EC and the level of monocyte and platelet adhesion assessed by phase contrast microscopy (n = 4). Data are mean ± SD. *P < .05, **P < .01, vs ADP only control (C, D) vs blood perfused in the absence of Dasatanib (E, F) using one‐way ANOVA followed by a Dunnett's post‐test

Flow cytometry analysis of platelets activated by ADP in the presence or absence of dasatanib provided the rationale for the observed inhibition of monocyte recruitment by the remaining adherent platelets (≈2%‐5% coverage in the presence of dasatanib). In both human and murine platelets, dasatanib inhibited the expression of platelet P‐selectin at ADP concentrations as high as 30 μmol/L (Figure 2A‐D).

In more stringent cell‐based models of vascular inflammation, we have previously identified TGF‐β1 as a potent agonist of VWF expression on EC. Indeed, TGF‐β1 can promote platelet adhesion and secondary leucocyte recruitment both in vitro^6^ and in vivo.^6^ Using an in vitro flow‐based assay, we observed that stimulation of human EC with TGF‐β1 (10 ng/mL) resulted in substantial adhesion of human platelets from flowing whole blood (Figure 2E), which in turn supported the secondary recruitment of monocytes from the flowing blood.

Importantly, addition of dasatanib (10 μmol/L) to blood prior to perfusion across activated EC, significantly reduced platelet coverage on TGF‐β1 stimulated EC. In turn, this had a marked effect on the efficiency of secondary recruitment of monocytes, resulting in a significant reduction in their adhesion (Figure 2E,F).

### 3.2 Genetic deletion of single SFK isoforms in mice alters platelet activation and inhibits the thrombo‐inflammatory recruitment of monocytes

**Figure 3:**
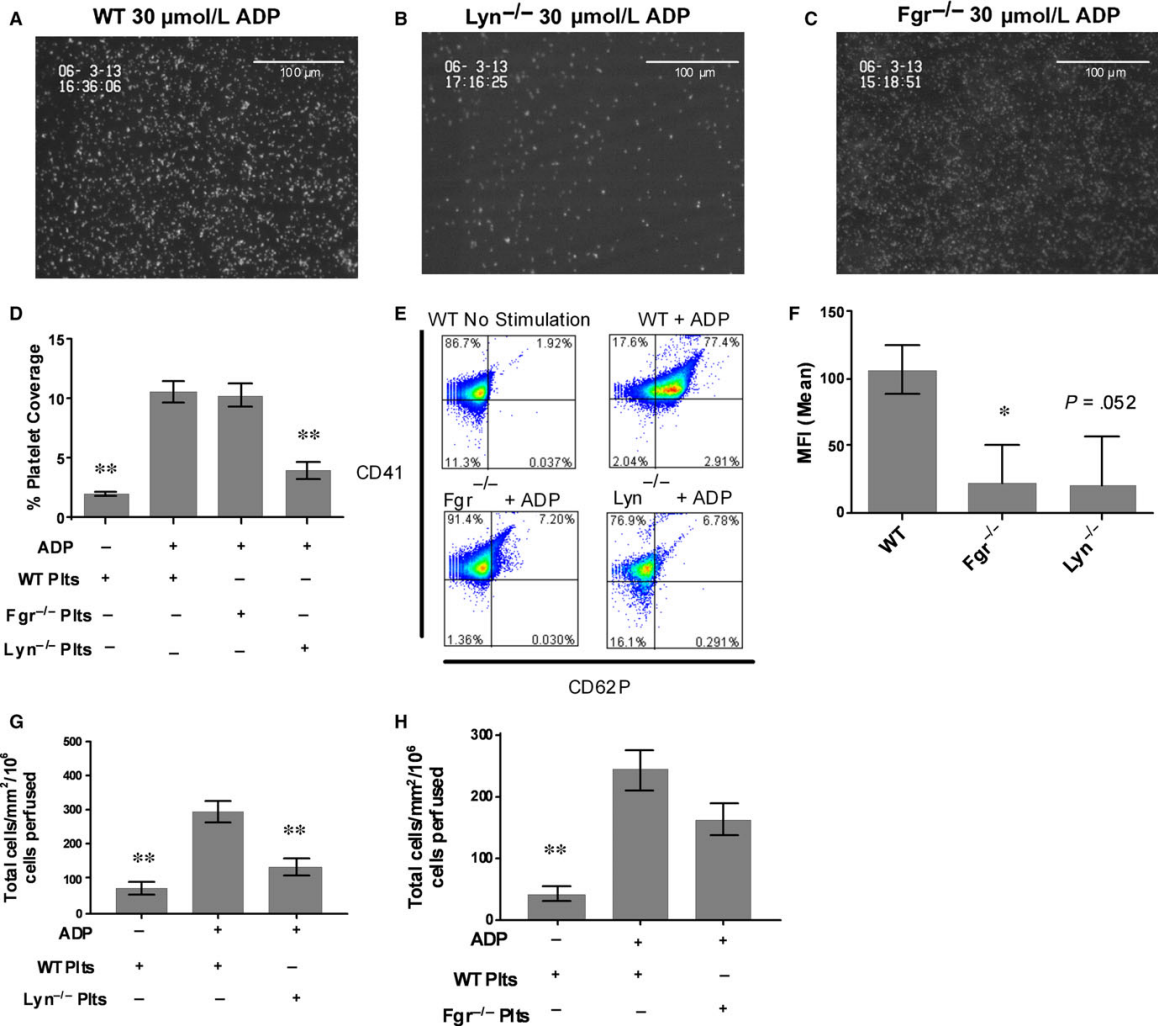
Mice deficient in Fgr or Lyn demonstrate decreased platelet activation, P selectin expression and leucocyte recruitment to VWF under flow conditions. A, Representative images of WT (B) Lyn^−/−^ or (C) Fgr^−/−^ platelets activated with 30 μmol/L ADP on a matrix of immobilized VWF in the presence of cells; (D) Average platelet coverage from flow (1000^−s^) on a matrix of immobilized VWF in the presence of 30 μmol/L ADP. The level of platelet adhesion was assessed by fluorescent microscopy. Data are mean ± SEM. compared to WT (C57Bl6) ADP positive controls (n = 15). E, Representative flow cytometry plot and (F) MFI ± SD of P‐selectin expression on WT, Lyn^−/−^ or Fgr^−/−^ platelets in response to ADP (n = 3). G, Number of WT monocytes adhering to Lyn^−/−^ or (H) Fgr^−/−^ platelets under flow conditions (100^−s^). The level of monocyte was adhesion assessed by phase contrast microscopy (n = 18). Data are mean ± SEM. Data are shown as mean ± SD. *P < .05, **P < .01 compared to the WT ADP positive control using one‐way ANOVA followed by a Dunnett's post‐test

Having demonstrated that the removal of redundant SFK had dramatic effects on the thrombo‐inflammatory recruitment of leucocytes, we tested whether platelets deficient in single SFK isoforms showed any deficit in thrombo‐inflammatory activity. Using whole blood taken from animals with a complete knock out of the most and least abundant SFK's in platelets (Lyn and Fgr, respectively), we found that platelets were readily recruited to and rolled upon immobilized VWF. The number of adherent platelets did not differ in magnitude in wild type or SFK knockout animals. However, upon super perfusion of ADP across rolling platelets, dramatic differences in platelet function were evident. Wild type platelets became rapidly activated and spread on the adhesive substrate forming microthrombi (Figure 3A,D). Lyn^−/−^ platelets did not become activated, and were washed from the system as they rolled on the VWF substrate (Figure 3B,D). Fgr^−/−^ platelets showed an intermediate phenotype, becoming stationary adherent on the VWF, but failing to fully activate, spread or form aggregates (Figure 3C,D).

The acquisition of such a profound phenotype in single SFK knockout animals prompted us to determine whether the expression of P‐selectin from α‐granules, which would be required the thrombo‐inflammatory secondary recruitment of monocytes, was also affected in animals deficient in a single SFK isoform. In both Lyn^−/−^ and Fgr^−/−^ platelets, P‐selectin expression was dramatically reduced in the presence of ADP (Figure 3E,F). Indeed, the levels of inhibition of degranulation (and therefore P‐selectin expression) were not dissimilar to that seen in wild type platelets pre‐treated with disatanib (Figure 2B,D).

Because the effects of knocking out single SFK isoforms on platelet function were so profound, we tested whether these extended to the secondary, thrombo‐inflammatory recruitment of monocytes. To do this we established populations of wild type, Fgr^−/−^ or Lyn^−/−^ platelets on immobilized VWF. Following activation by ADP, we perfused isolated murine monocytes across the activated platelets. The secondary recruitment of wild type leucocytes by ADP stimulated Lyn^−/−^ platelets was dramatically (>60%) reduced, when compared to recruitment by WT platelets (Figure 3G). A similar but less robust inhibition of leucocyte recruitment was evident when Fgr^−/−^ platelets were compared to wild type (Figure 3H).

The use of an ex vivo adhesion assay also allowed us to determine whether the loss of SFK signalling in leucocytes was redundant in the process of recruitment, as recently indicated by Kovacs et al^35^ In fact, when we used mononuclear leucocytes isolated from the Lyn^−/−^ or the Fgr^−/−^ strains, and measured the levels of their recruitment by wild‐type platelets, we observed significant reductions in the efficiency of secondary recruitment in both the Lyn^−/−^ and the Fgr^−/−^ deficient leucocytes, strongly implying that non‐redundant SFK pathways supporting thrombo‐inflammatory recruitment of monocytes were also operative in leucocytes (Supporting information Figure S4).

### 3.3 Genetic deletion of single SFK isoforms in mice dramatically reduces the burden of disease in the ApoE^*−/−*^ model of atherosclerosis

The efficiency with which single SFK knockouts could inhibit both platelet function and the secondary recruitment of monocytes in ex vivo assays leads to the important conclusion that such molecules represent therapeutic targets in thrombo‐inflammatory pathways of vascular disease. Such a supposition needed to be tested using integrated in vivo models of disease which would stringently probe the degree of redundancy in SFK signalling at a systemic level, and would also establish whether signalling pathways additional to SFK could support pathways of platelet and leucocyte activation in complex multicellular process supporting disease pathogenesis. In this context crossing the Lyn^−/−^ or Fgr^−/−^ knockout strains with the ApoE^−/−^ model of atherosclerosis provide an ideal test of this hypothesis. Moreover, using whole body SFK knockouts, these models have the benefit that they replicate the systemic effects (ie multi‐cellular targets) of pharmacological agents targeting specific SFK isoforms.

**Figure 4:**
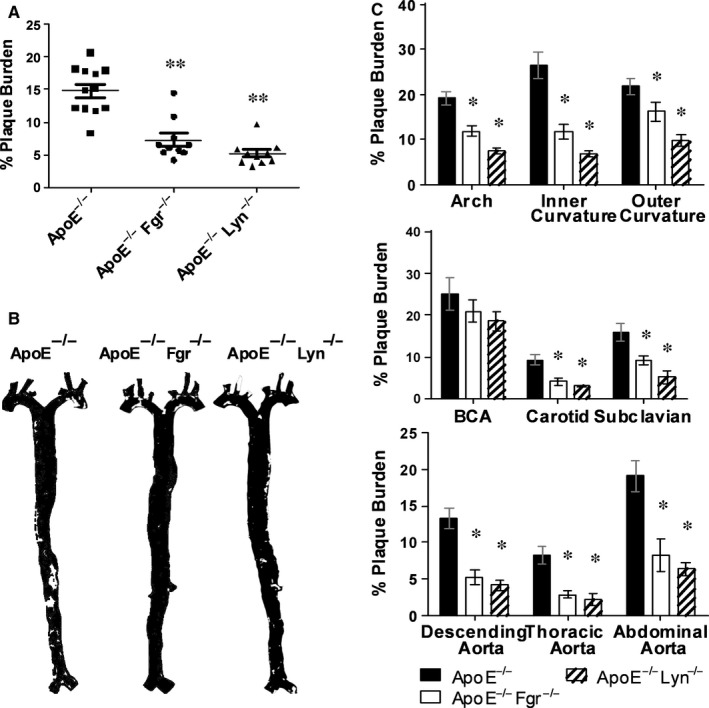
Decreased plaque burden in the aortas of ApoE^−/−^ mice on HFD for 6 wk after Fgr and Lyn abolition. A, Plaque burden in the aortas of Apoe^−/−^ animals or ApoE^−/−^ mice deficient in Fgr or Lyn assessed using analysis of oil red O staining after 6 wk of HFD (n = 10‐12). B, False colour masks depicting plaque burden in the aortas of Apoe^−/−^ animals or ApoE^−/−^ mice deficient in Fgr or Lyn. C, Site specific analysis of plaque burden within the aorta (n = 10‐12). Data are mean ± SEM *P < .05, **P < .01 *ApoE* ^−/−^ *Fgr* ^−/−^ *and ApoE* ^−/−^ *Lyn* ^−/−^ mice compared to *ApoE* ^−/−^ using one‐way ANOVA followed by a Dunnett (A) or Bonferroni (B) post‐test

**Figure 5:**
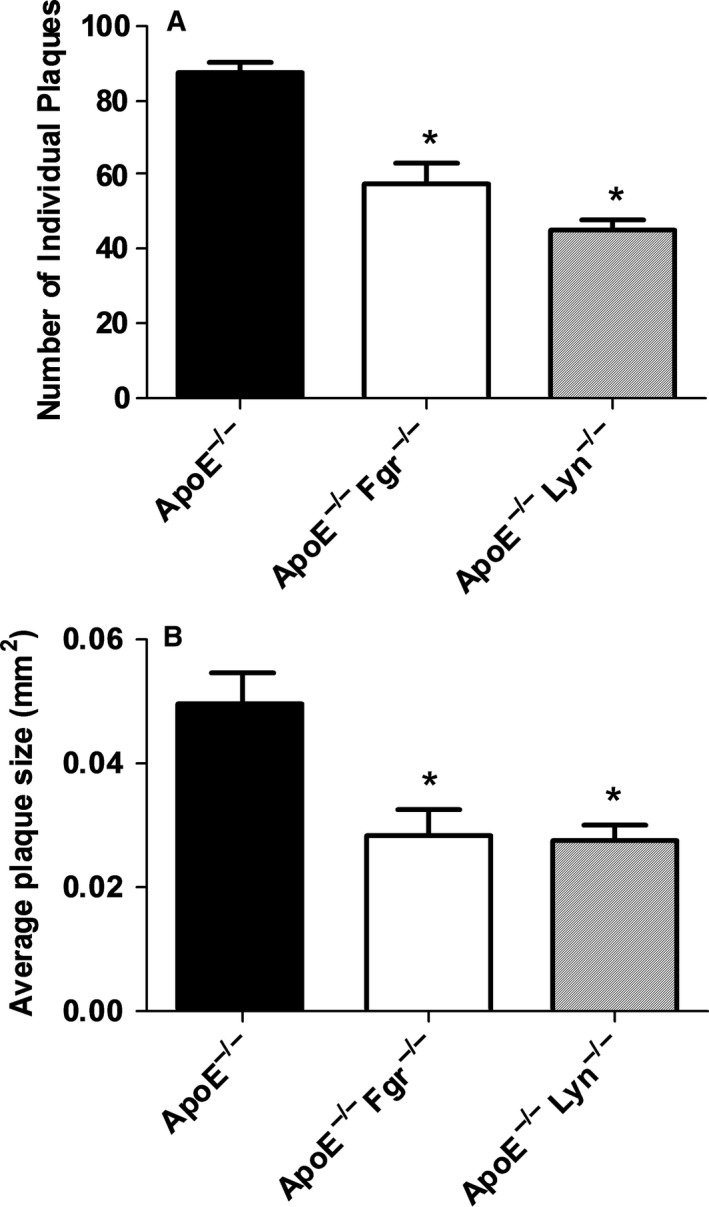
Decreased size and number of plaques in the descending aorta of ApoE^−/−^ mice on HFD for 6 wk after Fgr and Lyn abolition. A, B, *ApoE* ^−/−^ mice that were WT or deficient in Fgr or Lyn were placed on a HFD at 6 wk of age for a total of 6 wk. Total number of individual plaques (A) or average plaque size (B) in the descending aorta was analysed (n = 10‐12 per group). *P < .05, *ApoE* ^−/−^ *Fgr* ^−/−^ *and ApoE* ^−/−^ *Lyn* ^−/−^ mice compared to *ApoE* ^−/−^ using one‐way ANOVA followed by a Dunnett (A) post‐test

Total platelet count and percentage circulating peripheral blood lymphocytes (PBL), monocytes (Mono) and neutrophils (PMN) did not vary between *ApoE* ^−/−^ Lyn^−/−^ and ApoE^−/−^ Fgr^−/−^ deficient mice and *ApoE* ^−/−^, Lyn^*+/+*^ and Fgr^+/+^ controls (Supporting information Table S1). *ApoE* ^−/−^ animals fed a high fat diet (HFD) for 6 weeks demonstrated a significant level of plaque formation equivalent to ≈15% plaque coverage over the whole aorta (Figure 4A,B). Interestingly, the loss of either Lyn or Fgr led to a dramatic decrease in disease burden over the whole aorta (Figure 4A,B; >70% and 60% reduction in plaque coverage, respectively). Detailed analysis of the anatomically distinct regions of the aorta and the major branching arteries of the aortic arch, showed that disease was reduced uniformly across the arterial tree (Figure 4C). Interestingly, in both the Lyn^−/−^/*ApoE* ^−/−^ and Fgr^−/−^/*ApoE* ^−/−^ strains both the number and the average size (Figure 5A,B) of atheromatous lesions was significantly reduced. Finally, we observed no significant differences in the percentage of collagen content and cellularity in the plaque of all groups analysed indicating that although plaque size was sensitive to SFK knockout, the phenotype of the plaques did not vary (Supporting information Figure S5).

## 4 DISCUSSION

Direct evidence that thrombo‐inflammatory pathways are relevant to the development of vascular disease comes from murine models of atherosclerosis, where inhibition of platelet adhesion, or induction of thrombocytopenia, significantly reduced the burden of atheroma.^5^, ^36^, ^37^ In addition, instillation of activated platelets exacerbates arterial disease in such models.^5^ There is also direct evidence that platelet P‐selectin plays a role in plaque formation in the ApoE^−/−^ mouse.^38^, ^39^ Other studies demonstrate that platelet‐derived chemokines such as CCL5 can selectively recruit monocytes in these models.^40^, ^41^ In addition the preferential recruitment of monocytes to TGF‐β1 stimulated EC in vitro and in vivo is mediated by platelet bridges.^6^ TGF‐β1 promotes the expression of a matrix of VWF on the EC surface which recruits platelets from flowing blood. Upon platelet activation at the EC surface by ADP, monocytes are in turn recruited by platelet P‐selectin.^6^, ^42^ Experiments such as these, that show the marked effects of modulating thrombo‐inflammation, indicate that interruption of these pathways may have utility in regulating the cellular pathology of diseases such as atherosclerosis. Moreover, this could be an important target for such diseases, as thrombo‐inflammatory pathways may well fall without the control of more conventional therapies for regulating inflammation in other diseases.

The efficacy of knocking out a single SFK in such experiments is interesting, particularly so as it is not even necessary to target the most highly expressed SFK to modulate the activation of platelets in these physiological assays. In murine platelets, the predominant SFKs expressed in order of their abundance are Lyn, Src, Fyn and Fgr.^43^ Indeed the 2 SFK targeted in the current study (Lyn and Fgr) show nearly a 100‐fold differential in expression in platelets.^43^ Interestingly, the responses to activation of platelets from the Fgr^−/−^ and Lyn^−/−^ animals were distinct, demonstrating that these SFK regulate different pathways downstream of activation with ADP. However, it would appear that both pathways must be integrated for efficient platelet activation, spreading and P‐selectin expression and importantly, their functions are not redundant.

In the context of thrombo‐inflammatory pathways of monocyte recruitment in atheromatous disease, it appears that SFK play a critical role in disease progression. Undoubtedly, the most unexpected, and thus the most interesting aspect of our studies has been the ability to demonstrate that SFK signalling is not redundant when physiologically exacting models of platelet and leucocyte function are used to probe these pathways. Non‐redundant functions of SFK have previously been observed, for example in the central nervous system. However, in this study the activity of Fyn was involved in signal transduction from a single membrane receptor (NCAM‐140) feeding back on the process of neuronal migration.^20^ In the immune system and during an inflammatory response, multiple adhesion and activation receptors feed into the leucocyte trafficking process, and it is assumed that redundancy occurs due to the number of alternative routes by which recruitment can be facilitated. Thus, it is important to appreciate that even in a process involving multiple cell types (here, monocytes, platelets and endothelial cells), in a paradigm requiring multiple interactions between adhesion and activation pathways, the loss of function of a single SFK can have dramatic moderating effects on disease burden in integrated models of arterial disease. These benefits appear to be achievable without undue influence on the haemostatic and immune systems, as no significant bleeding phenotypes are reported in the single SFK knockout strains.^43^ Certainly, we observed no excess morbidity or mortality in our own SFK knockout cohorts over the duration of these experiments when compared to wild type or ApoE^−/−^ control animals. In addition, we observed no changes in plaque stability in these animals as collagen content and cellularity did not differ between the groups. This is interesting as it implies that targeting single SFK's therapeutically would not result in detrimental changes in plaque morphology that might lead to an increased risk of thrombotic complications. Thus, we believe that developing specific inhibitors to SFK isoforms is a rational approach to targeting disease pathways.

## COMPETING INTERESTS

None.

## ADDENDUM

M. J. Harrison conceived and performed experiments, analysed and interpreted the data, and co‐wrote the manuscript. M. Chimen conceived, performed experiments, analysed and interpreted the data. M. T. Hussain, A. J. Iqbal, Y. A. Senis, G. B. Nash, S. P. Watson organized and conducted the study, including analysis, data interpretation and critique of the manuscript. G. E. Rainger conceived, designed, organized and conducted the study, including analysis and interpretation of data, and co‐wrote the manuscript.

## References

[1] Ross R . Cell biology of atherosclerosis. Annu Rev Physiol. 1995;57:791‐804. 7778883 10.1146/annurev.ph.57.030195.004043

[2] Diacovo T , Roth S , Buccola J , Bainton D , Springer T . Neutrophil rolling, arrest, and transmigration across activated, surface‐adherent platelets via sequential action of P‐selectin and the beta 2‐integrin CD11b/CD18. Blood. 1996;88:146‐157. 8704169

[3] Butler LM , Metson‐Scott T , Felix J , et al. Sequential adhesion of platelets and leukocytes from flowing whole blood onto a collagen‐coated surface: requirement for a GpVI‐binding site in collagen. Thromb Haemost. 2007;97:814‐821. 17479193

[4] da Costa Martins P , van den Berk N , Ulfman LH , Koenderman L , Hordijk PL , Zwaginga JJ . Platelet‐monocyte complexes support monocyte adhesion to endothelium by enhancing secondary tethering and cluster formation. Arterioscler Thromb Vasc Biol. 2004;24:193‐199. 14615387 10.1161/01.ATV.0000106320.40933.E5

[5] Huo Y , Schober A , Forlow SB , et al. Circulating activated platelets exacerbate atherosclerosis in mice deficient in apolipoprotein E. Nat Med. 2003;9:61‐67. 12483207 10.1038/nm810

[6] Kuckleburg CJ , Yates CM , Kalia N , et al. Endothelial cell‐borne platelet bridges selectively recruit monocytes in human and mouse models of vascular inflammation. Cardiovasc Res. 2011;91:134‐141. 21285294 10.1093/cvr/cvr040

[7] Rinder HM , Bonan JL , Rinder CS , Ault KA , Smith BR . Dynamics of leukocyte‐platelet adhesion in whole blood. Blood. 1991;78:1730‐1737. 1717069

[8] Baigent C , Blackwell L , Collins R , et al. Aspirin in the primary and secondary prevention of vascular disease: collaborative meta‐analysis of individual participant data from randomised trials. Lancet. 2009;373:1849‐1860. 19482214 10.1016/S0140-6736(09)60503-1 PMC2715005

[9] Eikelboom JW , Hirsh J , Spencer FA , Baglin TP , Weitz JI. Antiplatelet drugs: antithrombotic therapy and prevention of thrombosis: American College of Chest Physicians evidence‐based clinical practice guidelines. CHEST J. 2012;141(2_suppl):e89S‐e119S. 10.1378/chest.11-2293 PMC3278069 22315278

[10] Patrono C , Andreotti F , Arnesen H , et al. Antiplatelet agents for the treatment and prevention of atherothrombosis. Eur Heart J. 2011;32:2922‐2932. 22019823 10.1093/eurheartj/ehr373

[11] Husted S , Storey RF , Harrington RA , Emanuelsson H , Cannon CP . Changes in inflammatory biomarkers in patients treated with ticagrelor or clopidogrel. Clin Cardiol. 2010;33:206‐212. 20394040 10.1002/clc.20732 PMC6653411

[12] Quinn MJ , Bhatt DL , Zidar F , et al. Effect of clopidogrel pretreatment on inflammatory marker expression in patients undergoing percutaneous coronary intervention. Am J Cardiol. 2004;93:679‐684. 15019868 10.1016/j.amjcard.2003.11.048

[13] Gawaz M , Langer H , May AE . Platelets in inflammation and atherogenesis. J Clin Invest. 2005;115:3378. 16322783 10.1172/JCI27196 PMC1297269

[14] Boggon TJ , Eck MJ . Structure and regulation of Src family kinases. Oncogene. 2004;23:7918‐7927. 15489910 10.1038/sj.onc.1208081

[15] Lowell CA , Soriano P . Knockouts of Src‐family kinases: stiff bones, wimpy T cells, and bad memories. Genes Dev. 1996;10:1845‐1857. 8756343 10.1101/gad.10.15.1845

[16] Kawakami Y , Furue M , Kawakami T . Identification of fyn‐encoded proteins in normal human blood cells. Oncogene. 1989;4:389‐391. 2649850

[17] Lock P , Ralph S , Stanley E , Boulet I , Ramsay R , Dunn AR . Two isoforms of murine hck, generated by utilization of alternative translational initiation codons, exhibit different patterns of subcellular localization. Mol Cell Biol. 1991;11:4363‐4370. 1875927 10.1128/mcb.11.9.4363-4370.1991 PMC361298

[18] Yi TL , Bolen JB , Ihle JN . Hematopoietic cells express two forms of lyn kinase differing by 21 amino acids in the amino terminus. Mol Cell Biol. 1991;11:2391‐2398. 2017160 10.1128/mcb.11.5.2391-2398.1991 PMC359994

[19] Corey SJ , Anderson SM . Src‐related protein tyrosine kinases in hematopoiesis. Blood. 1999;93:1‐14. 9864140

[20] Beggs HE , Soriano P , Maness PF . NCAM‐dependent neurite outgrowth is inhibited in neurons from Fyn‐minus mice. J Cell Biol. 1994;127:825‐833. 7962063 10.1083/jcb.127.3.825 PMC2120232

[21] Grant SG , O'Dell TJ , Karl KA , Stein PL , Soriano P , Kandel ER . Impaired long‐term potentiation, spatial learning, and hippocampal development in fyn mutant mice. Science. 1992;258:1903‐1910. 1361685 10.1126/science.1361685

[22] Giagulli C , Ottoboni L , Caveggion E , et al. The Src family kinases Hck and Fgr are dispensable for inside‐out, chemoattractant‐induced signaling regulating beta 2 integrin affinity and valency in neutrophils, but are required for beta 2 integrin‐mediated outside‐in signaling involved in sustained adhesion. J Immunol (Baltimore, Md : 1950). 2006;177:604‐611. 10.4049/jimmunol.177.1.604 16785558

[23] Lowell CA , Fumagalli L , Berton G . Deficiency of Src family kinases p59/61hck and p58c‐fgr results in defective adhesion‐dependent neutrophil functions. J Cell Biol. 1996;133:895‐910. 8666673 10.1083/jcb.133.4.895 PMC2120842

[24] Suen PW , Ilic D , Caveggion E , Berton G , Damsky CH , Lowell CA . Impaired integrin‐mediated signal transduction, altered cytoskeletal structure and reduced motility in Hck/Fgr deficient macrophages. J Cell Sci. 1999;112:4067‐4078. 10547366 10.1242/jcs.112.22.4067

[25] Fagerholm S , Hilden TJ , Gahmberg CG . Lck tyrosine kinase is important for activation of the CD11a/CD18‐integrins in human T lymphocytes. Eur J Immunol. 2002;32:1670‐1678. 12115650 10.1002/1521-4141(200206)32:6<1670::AID-IMMU1670>3.0.CO;2-M

[26] Perez OD , Mitchell D , Jager GC , Nolan GP . LFA‐1 signaling through p44/42 is coupled to perforin degranulation in CD56+CD8+ natural killer cells. Blood. 2004;104:1083‐1093. 15113754 10.1182/blood-2003-08-2652

[27] Fitzer‐Attas CJ , Lowry M , Crowley MT , et al. Fcgamma receptor‐mediated phagocytosis in macrophages lacking the Src family tyrosine kinases Hck, Fgr, and Lyn. J Exp Med. 2000;191:669‐682. 10684859 10.1084/jem.191.4.669 PMC2195832

[28] Lowell CA . Src‐family kinases: rheostats of immune cell signaling. Mol Immunol. 2004;41:631‐643. 15220000 10.1016/j.molimm.2004.04.010

[29] Wu Y , Asazuma N , Satoh K , et al. Interaction between von Willebrand factor and glycoprotein Ib activates Src kinase in human platelets: role of phosphoinositide 3–kinase. Blood. 2003;101:3469‐3476. 12393736 10.1182/blood-2002-03-0806

[30] Ezumi Y , Shindoh K , Tsuji M , Takayama H . Physical and functional association of the Src family kinases Fyn and Lyn with the collagen receptor glycoprotein VI‐Fc receptor γ chain complex on human platelets. J Exp Med. 1998;188:267‐276. 9670039 10.1084/jem.188.2.267 PMC2212454

[31] Kim S , Kunapuli SP . Negative regulation of Gq‐mediated pathways in platelets by G12/13 pathways through Fyn kinase. J Biol Chem. 2011;286:24170‐24179. 21592972 10.1074/jbc.M110.212274 PMC3129198

[32] Arias‐Salgado EG , Lizano S , Sarkar S , Brugge JS , Ginsberg MH , Shattil SJ . Src kinase activation by direct interaction with the integrin β cytoplasmic domain. Proc Natl Acad Sci. 2003;100:13298‐13302. 14593208 10.1073/pnas.2336149100 PMC263791

[33] Cooke BM , Usami S , Perry I , Nash GB . A simplified method for culture of endothelial cells and analysis of adhesion of blood cells under conditions of flow. Microvasc Res. 1993;45:33‐45. 8479340 10.1006/mvre.1993.1004

[34] Bahra P , Nash GB . Sparsely adherent platelets support capture and immobilization of flowing neutrophils. J Lab Clin Med. 1998;132:223‐228. 9735928 10.1016/s0022-2143(98)90171-8

[35] Kovacs M , Nemeth T , Jakus Z , et al. The Src family kinases Hck, Fgr, and Lyn are critical for the generation of the in vivo inflammatory environment without a direct role in leukocyte recruitment. J Exp Med. 2014;211:1993‐2011. 25225462 10.1084/jem.20132496 PMC4172222

[36] Davi G , Patrono C . Platelet activation and atherothrombosis. N Engl J Med. 2007;357:2482‐2494. 18077812 10.1056/NEJMra071014

[37] Massberg S , Schurzinger K , Lorenz M , et al. Platelet adhesion via glycoprotein IIb integrin is critical for atheroprogression and focal cerebral ischemia: an in vivo study in mice lacking glycoprotein IIb. Circulation. 2005;112:1180‐1188. 16103235 10.1161/CIRCULATIONAHA.105.539221

[38] Collins RG , Velji R , Guevara NV , Hicks MJ , Chan L , Beaudet AL . P‐Selectin or intercellular adhesion molecule (ICAM)‐1 deficiency substantially protects against atherosclerosis in apolipoprotein E‐deficient mice. J Exp Med. 2000;191:189‐194. 10620617 10.1084/jem.191.1.189 PMC2195808

[39] Dong ZM , Brown AA , Wagner DD . Prominent role of P‐selectin in the development of advanced atherosclerosis in ApoE‐deficient mice. Circulation. 2000;101:2290‐2295. 10811597 10.1161/01.cir.101.19.2290

[40] Schober A , Manka D , von Hundelshausen P , et al. Deposition of platelet RANTES triggering monocyte recruitment requires P‐selectin and is involved in neointima formation after arterial injury. Circulation. 2002;106:1523‐1529. 12234959 10.1161/01.cir.0000028590.02477.6f

[41] von Hundelshausen P , Weber KS , Huo Y , et al. RANTES deposition by platelets triggers monocyte arrest on inflamed and atherosclerotic endothelium. Circulation. 2001;103:1772‐1777. 11282909 10.1161/01.cir.103.13.1772

[42] Tull SP , Anderson SI , Hughan SC , Watson SP , Nash GB , Rainger GE . Cellular pathology of atherosclerosis: smooth muscle cells promote adhesion of platelets to cocultured endothelial cells. Circ Res. 2006;98:98‐104. 16322482 10.1161/01.RES.0000198386.69355.87

[43] Severin S , Nash CA , Mori J , et al. Distinct and overlapping functional roles of Src family kinases in mouse platelets. J Thrombo Haemost. 2012;10:1631‐1645. 10.1111/j.1538-7836.2012.04814.x PMC4280098 22694307

