# Supplementary material for: Signalling through Src family kinase isoforms is not redundant in models of thrombo‐inflammatory vascular disease
Source: J Cell Mol Med. 2018 Jul 4;22(9):4317–27. doi: 10.1111/jcmm.13721 (PMC6111872; doi:10.1111/jcmm.13721)
Supplement: Supplementary file 1 [file JCMM-22-4317-s001.docx]

# Supplementary Figures

**A**

**B**

**C**

**D**

**E**

**F**

**Supplemental Figure 1: Dasatinib decreases platelet aggregation in response to ADP.**

(**A-C**) Platelet rich plasma (PRP: 2×10^8^ml^-1^) prepared from Human, or (**D-F**) Murine (c57Bl6) blood was stimulated with 4µM (**A & D**), 10µM (**B & E**) or 30µM (**C & F**) adenosine diphosphate (ADP) ± 20µM Dasatinib (n=3). Data is shown as mean ±SD. * p<0.05 compared to the ADP positive control using one-way ANOVA followed by a Dunnett’s post test.

**A**

**B**

**Supplemental Figure 2: Fgr and Lyn abolition decreases platelet aggregation in response to ADP in murine platelets.**

(**A**) Platelet aggregation in response to different doses of ADP in murine PRP prepared from Fgr^-/-^ or (**B**) Lyn^-/-^ animals and compared to platelets from WT control (C57Bl6) mice. Data is mean ± SEM from n=4-13 mice per group.* p<0.05, vs WT (C57Bl6) control using one-way ANOVA followed by a Dunnett’s post test.

.

**
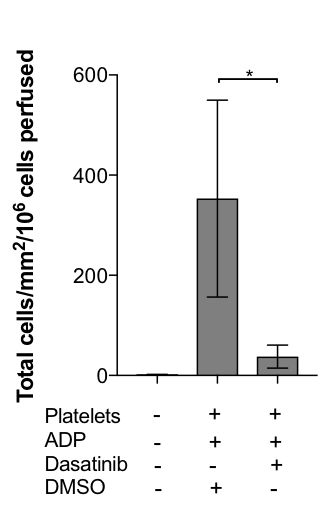
**

**Supplemental Figure 3: Dasatinib inhibits platelet activation and recruitment of neutrophils on immobilised VWF under flow conditions.** (**A**) The adhesion of human neutrophils and flowed across a monolayer of WT platelets bound to a matrix of immobilised VWF (100^-s^) (30µM ADP ± 20µM Dasatinib). The level of PBMC recruitment was assessed by phase contrast microscopy (n=3). Data is shown as mean ±SEM. *P<0.05 compared to ADP control using one-way ANOVA followed by a Dunnett’s post test.

**A**

**B**

**Supplemental Figure 4: PBMC deficient in Fgr or Lyn demonstrate decreased adhesion to WT platelets under flow conditions.**

**(**A) The adhesion of PBMC isolated from Fgr, or (**B**) Lyn deficient mice, and flowed across a monolayer of WT platelets bound to a matrix of immobilised VWF (100^-s^). The level of PBMC recruitment was assessed by phase contrast microscopy (n=18). Data is shown as mean ±SEM. *P<0.05 compared to WT PBMC using one-way ANOVA followed by a Dunnett’s post test.


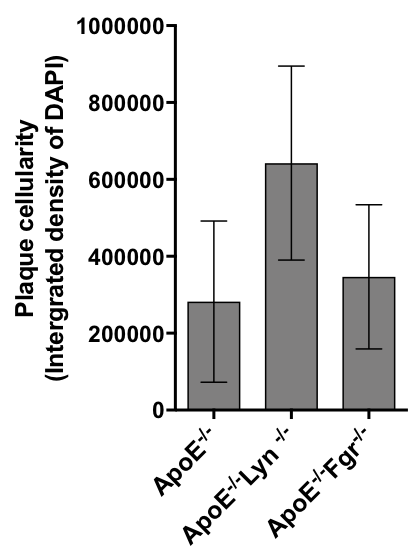

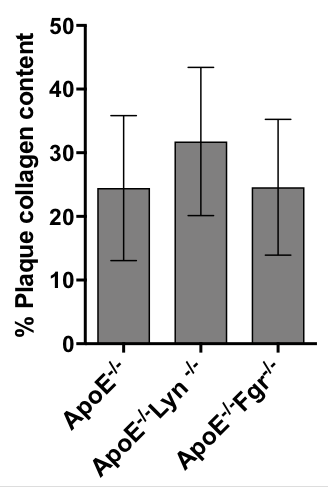


**A**

**B**

**Supplemental Figure 5: No changes in plaque collagen content and cellularity in the aortic root of ApoE^-/-^ mice on HFD for 6 weeks after Fgr and Lyn abolition.**

(**A**) Percentage of collagen content in the plaque of the aortic root of ApoE^-/-^ animals or ApoE^-/-^ mice deficient in Fgr or Lyn assessed using analysis of Van Gielsen staining after 6 weeks of HFD (n=6). (**B**) **A**) Cellularity in the plaque of the aortic root of ApoE^-/-^ animals or ApoE^-/-^ mice deficient in Fgr or Lyn assessed using analysis of DAPI intensity staining after 6 weeks of HFD (n=4). Data is mean ±SD *ApoE^-/-^ Fgr^-/-^ and ApoE^-/-^ Lyn^-/-^* mice were compared to *ApoE^-/-^* using one-way ANOVA followed by a Dunnett post test.
