# Supplementary material for: Signalling through Src family kinase isoforms is not redundant in models of thrombo‐inflammatory vascular disease
Source: J Cell Mol Med. 2018 Jul 4;22(9):4317–27. doi: 10.1111/jcmm.13721 (PMC6111872; doi:10.1111/jcmm.13721)
Supplement: Supplementary file 2 [file JCMM-22-4317-s002.doc]

**Supplemental Table 1. Blood counts, blood chemistry and weight profiles of mice on western Diet (6 wks)**

Males and females combined. ^*^ p<0.05 compared ApoE^-/-^ mice

| Mice | Total Plts  (K/μl) | Total WBC (K/µl) | PBL (%) | Mono (%) | PMN (%) | Start weight  (6 wks) | | End weight  (12 wks) | Total Weight Gain (g) | |
| --- | --- | --- | --- | --- | --- | --- | --- | --- | --- | --- |
| ApoE^-/-^  (n=13) | 744.7±  43.5 | 6.6±  0.8 | 58.1±  6.2 | 16.4±  4.5 | 19.0±  1.4 | | 19.4±  1.0 | 26.5±  1.7 | | 6.6±  0.8 |
| ApoE^-/-^ Fgr^-/-^  (n=12) | 810.8±  56.3 | *10.5±  1.0 | 64.8±  4.0 | 11.2±  1.3 | 20.6±  3.6 | | 20.4±  1.5 | 28.0±  1.6 | | 7.6±  1.4 |
| ApoE^-/-^ Lyn^-/-^  (n=10) | 620.6±  80.3 | 5.0±  0.8 | 63.6±  4.8 | 14.4±  4.2 | 18.0±  1.5 | | 20.7±  0.7 | 28.4±  1.6 | | 7.7±  1.1 |
